# New Oral Surgery Materials for Bone Reconstruction—A Comparison of Five Bone Substitute Materials for Dentoalveolar Augmentation

**DOI:** 10.3390/ma13132935

**Published:** 2020-06-30

**Authors:** Marcin Kozakiewicz, Tomasz Wach

**Affiliations:** Department of Maxillofacial Surgery, Medical University in Lodz, Pl. Hallera 1, 90–647 Łódź, Poland; marcin.kozakiewicz@umed.lodz.pl

**Keywords:** alveolar crest, augmentation, bone substitute material, intraoral radiograph, texture analysis

## Abstract

This article presents a comparison of bone replacement materials in terms of their ability to produce living bone image at the place of their implantation. Five bone replacement materials are compared (Osteovit—porous collagen, Cerasorb Foam—collagen scaffolding of synthetic β tricalcium phosphate, Osbone—synthetic hydroxyapatite, Endobone—deproteinized bovine-derived cancellous bone hydroxyapatite, and Cerasorb—synthetic β tricalcium phosphate). Intraoral radiographs are taken immediately after implantation and 12 months later. The texture analysis was performed to assess (texture index, TI) the level of structure chaos (entropy) in relation to the presence of longitudinal elements visible in radiographs (run length emphasis moment). The reference ratio of the chaotic trabecular pattern (Entropy) to the number of longitudinal structures, i.e., trabeculae (LngREmph), is 176:100 (i.e., 1.76 ± 0.28). Radiological homogeneity immediately after the implantation procedure is a result of the similar shape of its particles (Osbone, Endobone and Cerasorb) or radiolucency (Osteovit, Cerasorb Foam). The particles visible in radiographs were similar in the LngREmph parameters applied to the reference bone, but not in the co-occurrence matrix features. The TI for Osteovit during a 12-month follow-up period changed from 1.55 ± 0.26 to 1.48 ± 0.26 (*p* > 0.05), for Cerasorb Foam from 1.82 ± 0.27 to 1.63 ± 0.24 (*p* < 0.05), for Osbone from 1.97 ± 0.31 to 1.74 ± 0.30 (*p* < 0.01), and for Endobone from 1.86 ± 0.25 to 1.84 ± 0.25 (*p* > 0.05), The observed structure in the radiological image of bone substitute materials containing calcium phosphates obtains the characteristics of a living bone image after twelve months.

## 1. Introduction

Bone replacement materials (BRM) have been used in dentistry for decades [1]. They have undergone a long evolutionary process and are now most commonly used in periodontology [2], endodontic surgery [3], pre-prosthetic treatment [4,5] and oral implantology in the broadest sense of the term [6]. Different forms of calcium phosphates are most common. Initially, they were natural and synthetic hydroxyapatite [7], but nowadays calcium triphosphates and their mixtures with hydroxyapatite are used. To increase their osteoconductivity, a high degree of porosity is now common [8].

The purpose of their use is to reconstruct the alveolar ridge. Hence, dentists aim to reconstruct the bone in place of the defect. The best material would be one that regenerates a living bone rather than healing into the jawbone as a biologically inert substitute for the organism. This leads to the conclusion that, after a period of regeneration, the biomaterial should disappear, leaving a regenerated bone structure. If it is considered that the basic additional diagnostic test used by dentists is a radiograph, then it would be desirable to find on these radiographs this particular structure of living bone. How can a structure be defined in radiological imaging?

A possible method is to look for structure features [9,10]. This is not easy, as information on bone density or dimensions is most often searched for by the dentist. One would have to convert this information from quantitative data (millimeters) to qualitative data (bone structure). Fortunately, this is not the case, as there are methods for the quantitative (numerical) description of structure features, including texture analysis [11,12,13,14,15,16]. The authors chose entropy and long run emphasis moment based on experience from previous research where this feature has proved to be effective for the evaluation of bone healing processes.

The aim of this study is to objectively compare selected microstructure bone replacement materials using texture feature analyses of 2D X-ray images, in terms of their ability to produce living bone image at the place of their implantation.

## 2. Materials and Methods

The study was approved by the University Ethical Committee RNN/485/11/KB. A total of 263 patients (111 males and 152 females) aged between 17 and 75 years (48 ± 12 y) were included. All patients underwent surgical procedures prior to dental implant insertion: sinus lift (129 patients), jaw cyst removal (47 patients), tooth extraction (102 patients). Patients were divided into 5 groups depending on used material: Osteovit—an absorbable collagen (B Braun, Carl-Braun-Strasse 1, Melsungen, Hessen, Germany), Cerasorb Foam—an absorbable porous collagen scaffolding of synthetic β tricalcium phosphate (Curasan, 1768 Heritage Center Drive, Suite 201, Wake Forest, NC, USA), Osbone—a synthetic hydroxyapatite, 1–2 mm particles (Curasan, 1768 Heritage Center Drive, Suite 201, Wake Forest, NC, USA), Endobone—a deproteinized bovine-derived cancellous bone hydroxyapatite, 1–2 mm particles (Zimmer Biomet Dental, 4555 Riverside drive, Palm Beach Gardens, FL, USA), and Cerasorb—a synthetic β tricalcium phosphate, 1–2 mm particles (Curasan, 1768 Heritage Center Drive, Suite 201, Wake Forest, NC, USA). Normal trabecular bone was established as the reference group named “Bone”.

Inclusion criteria: two dimensional x-rays, a normal level of parathormone, a normal level of thyrotropin, a normal level of calcium in serum, a normal level of glycated hemoglobin, a normal level of vitamin D3 and normal densitometry. Exclusion criteria: lack of laboratory tests, defected radiographs (in the visual assessment of the author), complications after surgery and the need for bone grafting. There was no exclusion criteria taking into account general diseases other than those revealed in the above laboratory tests. The limitation of the study is that texture analyses do not reveal cellular microstructure of regenerated tissue, and clinicians should remember that this study is based on radiological analyses supported by statistical tests do not show the exact histological microstructure of tissues.

All intra-oral radiographs were taken during a typical clinical follow-up: starting on the day of surgery, directly after surgery (00M, 263 patients), and 12 months after surgery (12M, 177 patients). To take an intra-oral radiograph, a Digora Optime system of radiography was used (SOREDEX, Helsinki, Finland). The radiographs were taken in a standardized way [17] and with the strictly determined technical parameters: 7 mA, 70 mV an 0.1 s (Focus apparatus—Instrumentarium Dental, Tuusula, Finland). To make sure that the radiographs were repeatability, the authors used positioners (90° angle of X beam to the surface of phosphor plate). All radiographs were analyzed in MaZda software version 4.6, invented by the University of Technology in Lodz [18]. The main goal of this software is texture analysis and it allows evaluations of texture parameters in digital radiographs. In this study, 440 intraoral radiographs were analyzed.

**Figure 1:**
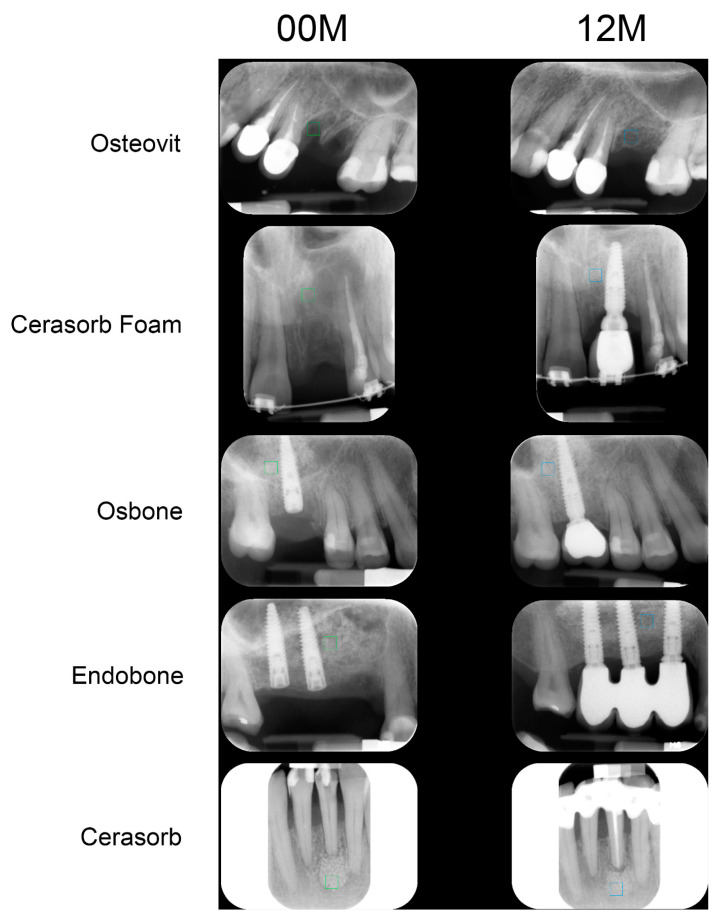
Examples of analyzed intra-oral radiographs (directly after implantation—00M, one year after surgery—12M). The exemplary regions of interest are marked in green and blue.

**Figure 2:**
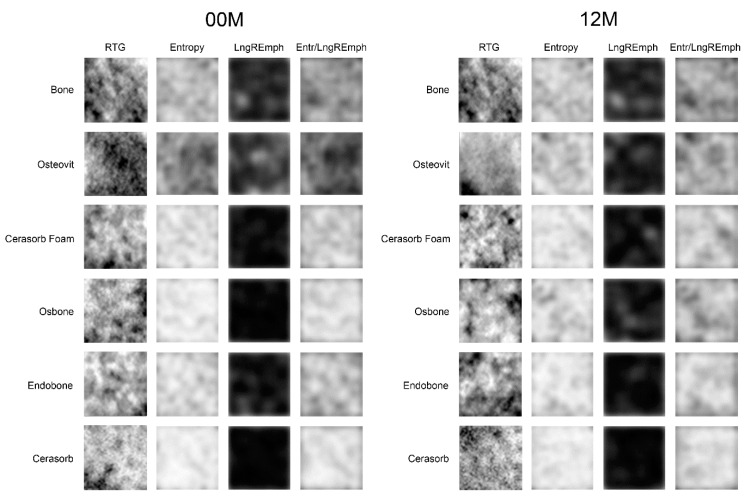
Investigated material (reference bone and five bone substitutes) and method of evaluation. Sample regions of interest in radiographs are named ROI. The following columns (Entropy, LngREmph, Entr/LngREmph) show a map of feature distribution in ROI.

The first step was to load the X-ray image to MaZda in a bitmap file format. Then, the ROIs (regions of interest) were marked with approximately 2500 pixels for bone area as well for bone substitute material area. The ROIs were normalized (μ ± 3σ) to share the same mean and standard deviation of grey level inside the ROI (where μ and σ denote the mean and standard deviation of registered optical density, respectively) (Figure 1 and Figure 2).

The map of the feature shows the intensity of the feature in ROI. The more white the pixel is, the more intense the feature is at this point.

Selected image features (Entropy from the co-occurrence matrix, and Long run emphasis moment from the run-length matrix) in ROIs were calculated for the reference bone and for the substitute material:

(1) $$Entropy=-\overset{Ng}{\underset{i=1}{\sum }}\overset{Ng}{\underset{j=1}{\sum }}p\left(i,j\right)log(p\left(i,j\right)$$

where Σ is sum, *Ng* is the number of levels of optical density in the radiograph, *i* and *j* are optical density of pixels 5 pixels apart from one another, *p* is probability, log is decimal logarithm [5].

(2) $$LngREmph=\frac{\sum_{i=1}^{Ng}\sum_{k=1}^{Nr}k^{2}p\left(i,k\right)}{\sum_{i=1}^{Ng}\sum_{k=1}^{Nr}p\left(i,k\right)}$$

where Σ is sum; *Nr* is the number of series of pixels with density level *i* and length *k*; *Ng*—number of levels for image density (8 bits i.e., 256 grey levels); *Nr*—number of pixel in series; *p* is probability [13,14]. These three equations were subsequently used for the texture index construction. Finally, the texture index, which represents the ratio of the measure of the diversity of structure observed in the radiograph to the measure of the presence of uniform and fine longitudinal structures, was calculated:

(3) $$Texture\;index=\frac{Entropy}{LngREmph}=\frac{(-\sum_{i=1}^{Ng}\sum_{j=1}^{Ng}p\left(i,j\right)log\left(p\left(i,j\right)\right))\sum_{i=1}^{Ng}\sum_{k=1}^{Nr}p\left(i,k\right)}{\sum_{i=1}^{Ng}\sum_{k=1}^{Nr}k^{2}p\left(i,k\right)}$$

The confirmed normal distribution features of the texture were compared by t-Student test, and the medians of the determined non-normal distribution features were compared by the Mann–Whitney (Wilcoxon) W-test. Multifactorial ANOVA for the detection of the source of variability regarding surgical procedure and type of material as factors was applied. Comparisons between different bone-replacement materials were carried out with one-way ANOVA or the Kruskal–Wallis test depending on the presence of normal distribution. When *p* < 0.05, it was assumed that the difference was statistically significant.

## 3. Results

The texture alterations over time are minor and involve Cerasorb Foam and Osbone (Table 1). Osbone’s result is influenced by the increase in the LngREmph of the component in the presence of constant entropy of the implantation site structure. In the case of materials containing collagen (Osteovit and Cerasorb Foam), a lower or higher decrease in the index Entr/LngREmph is observed due to the increase in entropy of the implantation site. In Cerasorb as well Endobone, the maintenance of the unchanged texture index is linked to a decrease in the value of entropy over 12 months and a slightly higher decrease in the LngREmph (Table 2). Ultimately, the ratio of these components will remain the same.

**Figure 3:**
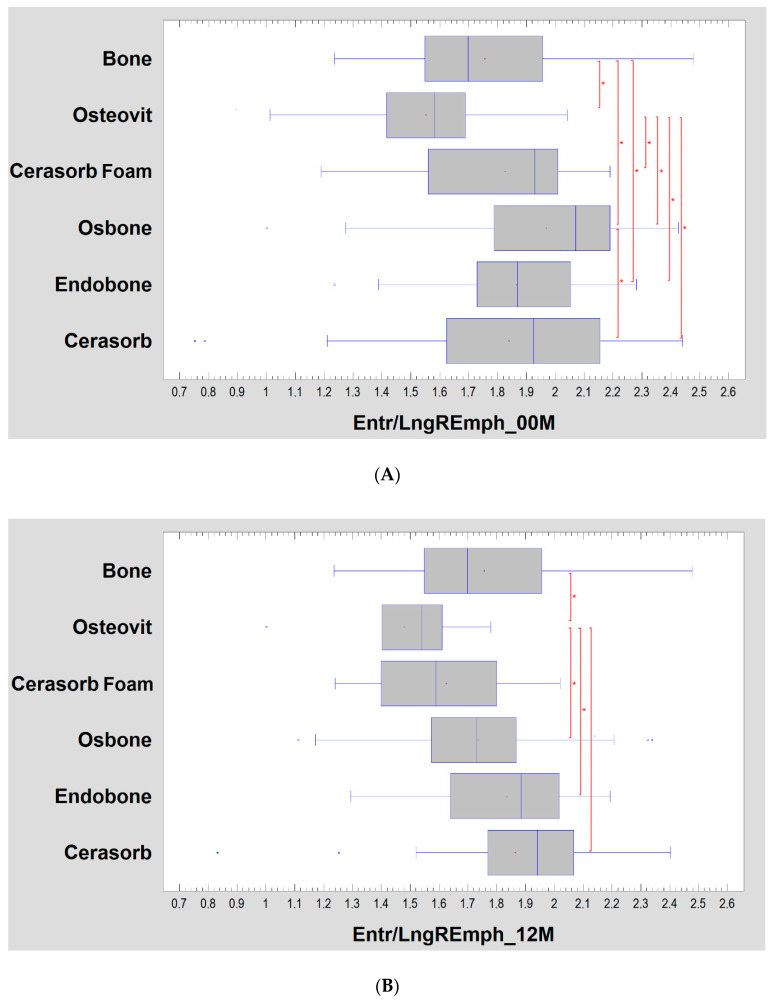

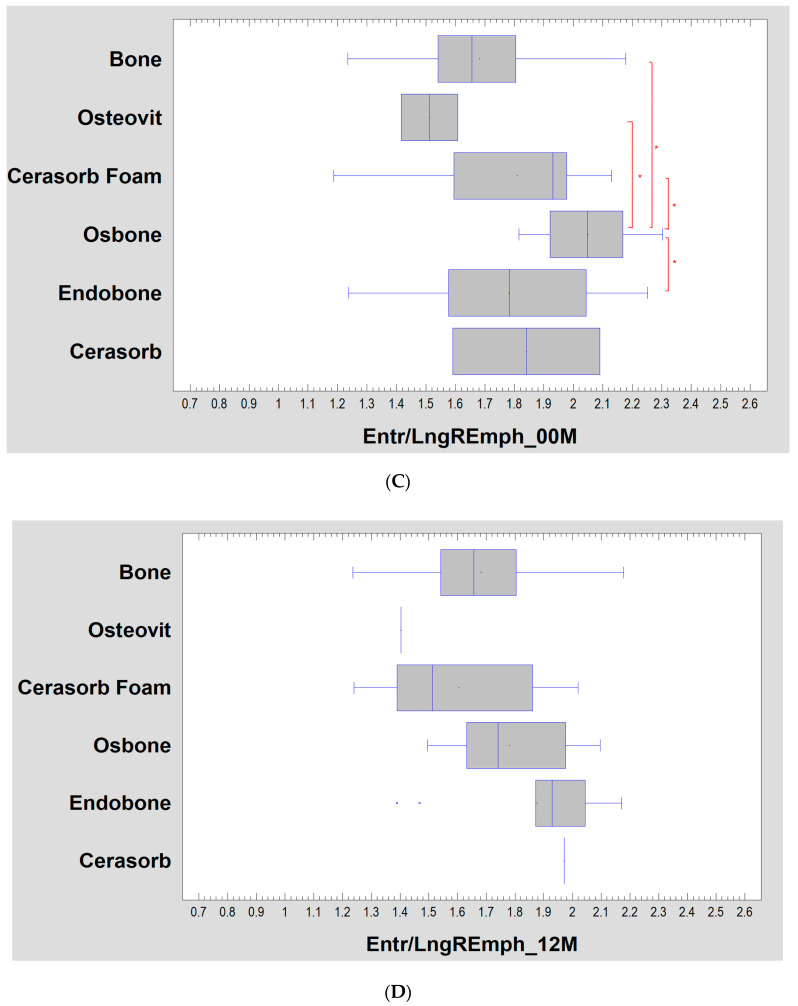
(**A**) Bone substitute-dependent alteration of radiotexture defined by the texture index in the implantation site. Observation immediately post-operation. Asterisks indicate a statistically significant difference (*p* < 0.05). (**B**) Bone substitute dependent-alteration of radiotexture defined by the texture index in the implantation site. Observation 12 months post-operation. Asterisks indicate a statistically significant difference (*p* < 0.05). (**C**) Bone substitute-dependent alteration of radiotexture defined by the texture index in the implantation site in a group of women >50 years old. Observation immediately post-operation. Asterisks indicate a statistically significant difference (*p* < 0.05). (**D**) Bone substitute-dependent alteration of radiotexture defined by the texture index in the implantation site in a group of women >50 years old. Observation 12 months post-operation. No statistically significant differences found.

Texture alterations depending on the implanted biomaterial are more pronounced (Figure 3A,B). The structure of biomaterials (00M) revealed by X-rays (or its invisibility in the case of Osteovit) is manifested by a higher texture index value for Endobone and Cerasorb than in the reference bone. All biomaterials show a higher index than Osteovit (test statistic = 37.5; *p* < 0.001). The separation of the group of postmenopausal women (>50 y.o.) slightly changed the results observed here, i.e., in the study after 12 months, the differences between the groups disappeared (Figure 3C,D).

The differences in the radiological image decreased after twelve months of healing. A weak Osteovit result remains, which differs significantly from the reference bone as well as Osbone, Endobone and Cerasorb (test statistic = 12.4; *p* < 0.05).

The simple regression shows the results of fitting a double reciprocal model to describe the relationship between Entr/LngREmph_12M and Entr/LngREmph_00M. There is a relation (*p* < 0.001). The equation of the fitted model is:

(4) $$Entr/LngREmph\_12M=\frac{1}{\left(0.209+\frac{0.665}{Entr/LngREmph\_00M}\right)}$$

**Figure 4:**
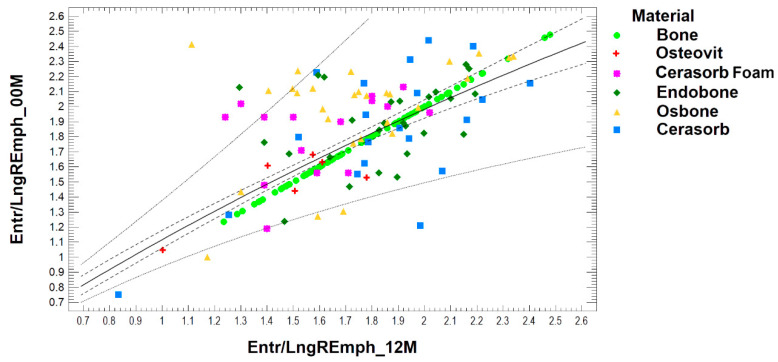
Dependence of the observed radiological texture at the site of biomaterial implantation created after twelve months of healing on the appearance of the radiological image of the recent biomaterial implanted. Simple regression plot calculated for all collected data. Note: solid line—regression plot; dotted line—prediction limits; dash line—confidence limit for *p* < 0.05.

The R^2^ statistic indicates that the fitted model explains 53% of the variability in Entr/LngREmph_12M. The correlation coefficient equals 0.73, indicating a moderately strong relationship between the variables. A regression plot and detailed results obtained for the tested bone substitute materials are presented in Figure 4.

**Figure 5:**
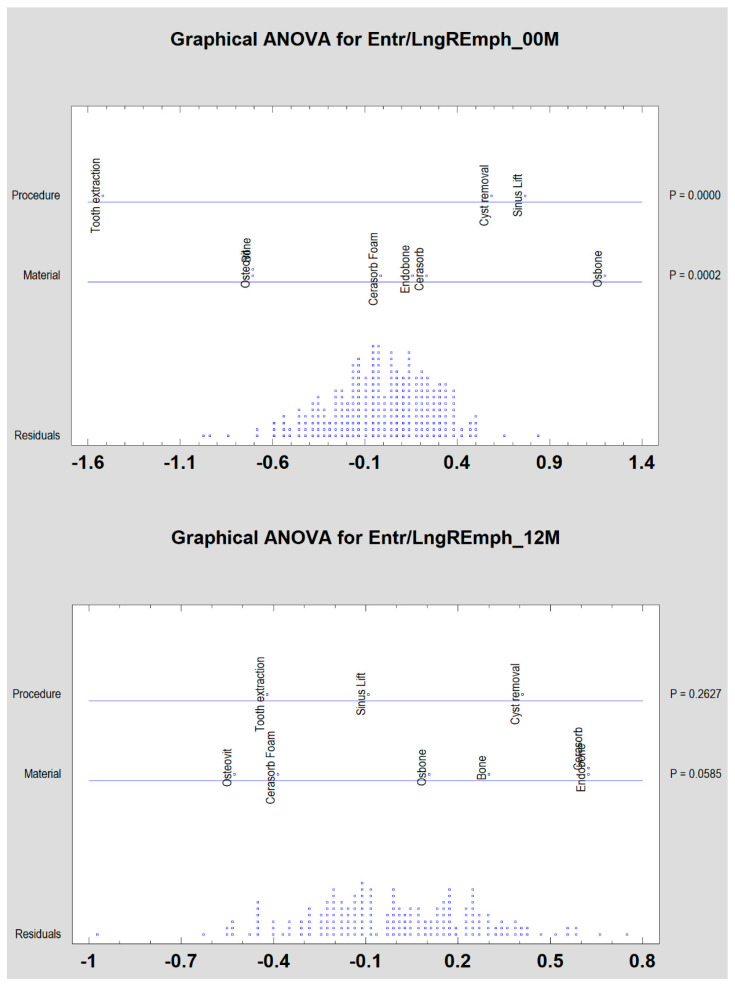
Bone substitute-dependent alteration of radiotexture defined by the texture index in the implantation site depending on surgical procedure. Observation immediately (00M) and 12 months post-operation (12M).

Both the types of surgical procedure performed and the type of bone substitute material used cause significant differentiation among the examined patients (*p* < 0.05) only immediately post-operation (Figure 5). Later on, these differences disappeared.

For easier understanding of the essence of the calculated results for the appearance of the analyzed image, Appendix A at the end of the text was prepared.

## 4. Discussion

There are not many radiological publications that focus on the behavior of implanted biomaterials. There seems to be some difficulty in objective radiological evaluation. The assessment of optical density in CBCT (cone beam-computed tomography) is not easy due to the impossibility of using the Hounsfield scale. The assessment of alveolar ridge dimensions [19], on the other hand, does not mention the presence of living bone [20]. Furthermore, the presence of living hard tissue allows for long-term clinical success. The presence of bone replacement granules in the alveolar ridge only reduces the volume of living bone. Usually, most of the augmentation site is filled with biomaterial and not bone [21]. This is especially true for hydroxyapatite (Endobone, Bio-Oss, InduCera, HA-Biocer, Osbone) [22], but it is also observed, although to a lesser extent, in tricalcium phosphates [23,24], such as Cerasorb, Bone Ceramic or ChronOS. Therefore, the use of materials with collagen scaffolding and filled with tricalcium phosphate (Cerasorb Foam) appears promising. Cerasorb Foam was much more similar (taking into account texture analysis) to the reference bone than other substitution materials. Following the observation period, we can assume that more living bone (which means less material granules) is in this material.

Intraoral radiography is widely used for evaluating the level and pattern of alveolar bone destruction. It is simple to acquire and has a relatively low cost and low radiation dose [25]. The effect of contrast-adjustment-based enhancement filters does not increase the overall diagnostic accuracy of periapical lesions [26,27,28,29]. Attempts are made to describe qualitatively the cancellous structure in the alveolar process in the intraoral images. A total of 26 bone features that can be counted [9] by means of labor-intensive image processing were determined. A related study [30] describes 25 (partly different features) variables of bone cancellous anisotropy in two-dimensional images which help to establish the trabeculae orientation. Such a second-order feature analysis may reveal more information about bone condition than just an assessment of optical density [31] or dimensions [19].

The bone structure surrounding the site of biomaterial introduction in women over fifty years old is different than in the entire population studied. However, observing Figure 2, one can comment that these differences are not extremely large. The graphs for the whole examined population and samples of women have a similar layout for the period immediately after surgery. The situation changes slightly after twelve months. The assessment is disturbed by small groups: Osteovit and Cerasorb. Unfortunately, the diversity observed in the study covering the whole population originates from these two sources.

If the impact of the surgical procedure and the biomaterial used is assessed, it can be seen that the texture of the implantation site defined as the ratio of entropy to the number of longitudinal structures detected in the X-ray test is identical after the healing period. This is observed after 12 months, although, the results were significantly different for the tooth extraction sites versus cyst removal/sinus lift (*p* < 0.0001) immediately after the implantation of the biomaterial.

The radiological homogeneity immediately after the implantation procedure is a result of the similar shape of its particles (Osbone, Endobone and Cerasorb) or radiolucency (Osteovit, Cerasorb Foam). The particles visible in radiographs may be similar in respect to applied run, that is, the length matrix parameter to the reference bone [23], but not in co-occurrence matrix features [24]. Hence, we propose the notion of combining two texture characteristics into one index: “LngREmph” derived from the run length matrix and “Entropy” derived from the co-occurrence matrix.

This study shows that the ratio of the chaotic cancellous bone pattern (Entropy) to the number of regular structures, i.e., trabeculae (LngREmph), is 176:100. This demonstrates a slight superiority in the diversity of the structure over its arrangement in the bone trabeculae (texture index equals 1.76 ± 0.28). If the number of trabeculae increases (higher LngREmph) or the building of microstructures is more homogenous (lower Entropy), the texture index decreases in value, corresponding to a compact bone. Alternatively, in the case of the bone substitute materials tested here, it would be a result of the rapid biomaterial resorption (ahigh number of dark pixel series, i.e., higher LngREmph) together with the lack of bone structure. This may be closer to the bone scar than the compacted bone (low entropy but in dark areas). Unfortunately, the LngREmph feature does not differentiate between light or dark series of pixels. This is the issue that remains with a simple collagen implantation (Osteovit).

Both of these situations can be considered to be clinically unfavorable because, on the one hand, the placement of dental implants into too compact bone is undesirable and, on the other, the existence of a bone scar makes the placement of the implant impossible.

It is also worth considering what the increased value of the texture index means. It indicates the existence of a very fine trabecular or even granular structure (low LngREmph value) with the coexistence of high chaos in its arrangement (high entropy). It would be difficult to find any positive interpretation of this state. One can only think of the lack of proper bone, e.g., failure of guided tissue regeneration (like in case of Osteovit here) or osteoporosis.

Osteovit is a clear collagen material that does not have enough mechanical endurance in bone regeneration sites. Collagen blended with synthetic β tricalcium phosphate revealed slightly higher mechanical properties (Cerasorb Foam). It is shown that it can obtain pure collagen from species other than bovine sources, e.g., marine collagen. Another important material used in controlled bone regeneration (GBR) is chitosan. It can be used as a main component of GBR barrier membranes or as a carrier of bone distal growth factors [32,33]. Incorporated chitosan increases the biological properties of algae-derived bone substitute materials to similar a similar level with that of bovine hydroxyapatite [34]. This material is novel and interesting, but a slower level of resorption of bone substitute materials is required when taking into account the results of our research.

The observed decrease in the index Entr/LngREmph (*p* < 0.01) for Osbone is caused by an increase in the LngREmph component, which indicates long dense structures appearing among un-resorbed particle of the bone substitute material. This is the strongest evidence for osteoconductivity in this experiment.

In the case of the material containing collagen, Cerasorb Foam, the results (*p* < 0.05) indicate the growth of a new bone, which, however, matures into a cancellous form. It simply fills the bone defect, reducing the chaotic structure (background trabeculae), i.e., the value of entropy. A similar mechanism may explain the observed alterations to the Cerasorb structure. A decrease in entropy (cancellous bone) may be responsible for ingrowth between granules of bone substitute material of a new bone that has been mineralized. At the same time, there is no resorption of Cerasorb within 12 months, which is responsible for a noticeable decrease in the LngREmph component. The structure is hard but with limited vascularization (due to the remains of biomaterial).

In the annual follow-up, the collagen component of Cerasorb Foam is clearly visible, i.e., the place of implantation is similar to the effect of collagen material (Osteovit). However, the augmentation result is successful to the extent that the difference with classical granular biomaterials (Osbone, Endobone and Cerasorb) cannot be detected. Cerasorb Foam is also similar to the reference bone too. The reduced value of the texture index observed in absolute values over one year indicates the need for further observation of the effectiveness of inorganic materials suspended in a collagen base (Cerasorb Foam), despite their excellent handling in a bone defect—even in a two-walled cavity.

Osbone (synthetic) and Endobone (bovine) are hydroxyapatites. This chemical compound has a long medical history as the component naturally occurs in human bones. They are generally recognized as slow resorbable materials [3]. The bone substitute remnants are found in implantation sites even after many years. On the opposite side are tricalcium phosphates, especially highly porous beta-phase materials like Cerasorb. The resorption rate is 6 months, but it is also possible to find some remnants later in the augmentation site.

The bone formation process starts with the absorption of proteins [35,36], the rate of this process depends on the microstructure of the material [37]. The differences in material structure may have a significant impact on protein adhesion and subsequent cell adhesion. The porosity of the bone-replacement material with different pore diameters and variable granule size is used to create a scaffold that provides a good structure for osteoconduction. The attachment phase is the next stage of new bone formation. Physical and chemical interactions take place in this stage [36]. Next, osteoblast adhesion appears, and this is known as the adhesion phase [37]. Interaction between cells is conditioned by physical and chemical features [23] of the surface and also, most importantly, the surface topography size [38], the shape [38,39] and the surface texture [40]. The above-described required properties of the bone substitute materials unfortunately are not enough. Osteovit, for example, has good adhesion properties for cells, but resorption of this material become too fast, and this is perhaps the reason why the required dimensions of regenerated regions are insufficient, and why ingrowth of soft tissue is too fast. Subsequently, this may explain why there is no living bone in texture analyses. Moreover, the microstructure of Osbone and Endobone, for example, enables good adhesion, but its resorbability index is higher and living bone occurs much more often.

Scaffold creation is a BRM feature. This can be visually evaluated and measured as image texture. The products from scaffold disintegration may then become the substrate for new bone deposition. During the resorption of the bone substitute, Ca^2+^ and PO_4_^3−^ ions are formed here. Newly formed connective tissue is mineralized due to these ions and becomes a new bone. After the one-year healing period, the observed similarity in the bone substitute materials (Osbone, Endobone) is a result of the ability of normal bone formation on the hydroxyapatite scaffold. The osteoconduction potential of tricalcium phosphate (Cerasorb) appears after significant resorption of granules, and this lead to bone regeneration. The ultimate osteological effect of these three tested materials is the same: restitution ad integrum.

## 5. Conclusions

The observed structure in the radiological image of the implantation sites of bone substitute materials containing calcium phosphates obtains the characteristics of a living bone image after twelve months. A slower level of resorption has an impact on the maintenance of the bone regeneration region that determines similarity to bone tissue and relevant dimensions of bone volume after the 12-month healing process. Nevertheless, slow resorbing granules of bone substitute material can be found in the regenerated region without being replaced by living bone tissue. The only exception in this experiment is pure collagen, which does not create the correct trabeculation of the alveolar crest—probably because of its high level of resorbability. Using the materials Osbone and Endobone as above-tested lead to good and predictable clinical outcomes, while Cerasorb needs additional time in regard to the healing process. Thanks to texture analyses, clinicians can predict whether an implantation is in living bone and determine what the necessary conditions will be for osteointegration between an implant and bone.

## Figures and Tables

**Table 1:** Time dependent alteration of the texture index (Entr/LngREmph) in the implantation site.

Material	00M	12M	Significance
Bone	1.76 ± 0.28	1.76 ± 0.28	n.s.
Osteovit	1.55 ± 0.26	1.48 ± 0.26	n.s.
Cerasorb Foam	1.82 ± 0.27	1.63 ± 0.24	*p* < 0.05
Osbone	1.97 ± 0.31	1.74 ± 0.30	*p* < 0.01
Endobone	1.86 ± 0.25	1.84 ± 0.25	n.s.
Cerasorb	1.84 ± 0.41	1.86 ± 0.36	n.s.

Abbreviations: 00M—immediately post-operationally, 12M—twelve months post-operationally, n.s.—not significant.

**Table 2:** Time dependent alteration of the entropy and LngREmph in the implantation site.

Material	Entropy		LngREmph	
	00M	12M	00M	12M
Bone	2.70 ± 0.18	2.70 ± 0.18	1.56 ± 0.21	1.56 ± 0.21
Osteovit	2.67 ± 0.14	2.67 ± 0.10	1.76 ± 0.28	1.86 ± 0.36
Cerasorb Foam	2.79 ± 0.18	2.67 ± 0.20	1.54 ± 0.19	1.65 ± 0.27
Osbone	2.86 ± 0.12	2.80 ± 0.12	1.49 ± 0.24	1.65 ± 0.25
Endobone	2.88 ± 0.10	2.86 ± 0.09	1.57 ± 0.19	1.58 ± 0.19
Cerasorb	2.78 ± 0.18	2.72 ± 0.18	1.60 ± 0.47	1.52 ± 0.35

Abbreviations: 00M—immediately post-operationally, 12M—twelve months post-operationally.

## Appendix A

How to understand numerical measures of texture features in intra-oral radiographs. The complexity of the test patterns is not as great as that observed in clinical radiographs and, therefore, the textural index (Entr/LngREmph) values are lower than those in the main text.

**Pattern**	**Entropy**	**LngREmph**	**Entr/** **LngREmph**	**Pattern**	**Entropy**	**LngREmph**	**Entr/** **LngREmph**
	1.79	39.5	0.045		2.62	4.5	0.581
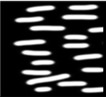
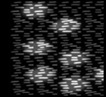
	1.84	21.0	0.087		2.79	2.6	1.069
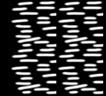
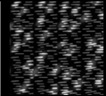
	2.03	6.3	0.320		2.80	2.1	1.341
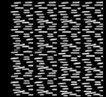
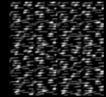
	1.92	35.2	0.054		2.41	5.6	0.429
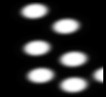
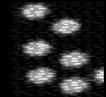
	2.55	5.9	0.432		2.73	2.6	1.046
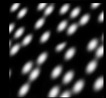
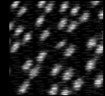
	2.55	3.7	0.689		2.77	2.1	1.327
